# Effect of Plasmid DNA Size on Chitosan or Polyethyleneimine Polyplexes Formulation

**DOI:** 10.3390/polym13050793

**Published:** 2021-03-05

**Authors:** J.F.A. Valente, P. Pereira, A. Sousa, J.A. Queiroz, F. Sousa

**Affiliations:** 1 CICS-UBI—Health Sciences Research Centre, Universidade da Beira Interior, Avenida Infante D. Henrique, 6200-506 Covilhã, Portugal; ppereira@fcsaude.ubi.pt (P.P.); angela@fcsaude.ubi.pt (A.S.); jqueiroz@ubi.pt (J.A.Q.); 2 CDRsp—Centre Rapid and Sustainable Product Development, Polytechnic Institute of Leiria, 2411-901 Leiria, Portugal; 3 CEMMPRE, Department of Chemical Engineering, University of Coimbra, Rua Sílvio Lima-Pólo II, 3030-790 Coimbra, Portugal

**Keywords:** chitosan, gene therapy, polyethyleneimine, polyplexes, transgene expression

## Abstract

Gene therapy could be simply defined as a strategy for the introduction of a functional copy of desired genes in patients, to correct some specific mutation and potentially treat the respective disorder. However, this straightforward definition hides very complex processes related to the design and preparation of the therapeutic genes, as well as the development of suitable gene delivery systems. Within non-viral vectors, polymeric nanocarriers have offered an ideal platform to be applied as gene delivery systems. Concerning this, the main goal of the study was to do a systematic evaluation on the formulation of pDNA delivery systems based on the complexation of different sized plasmids with chitosan (CH) or polyethyleneimine (PEI) polymers to search for the best option regarding encapsulation efficiency, surface charge, size, and delivery ability. The cytotoxicity and the transfection efficiency of these systems were accessed and, for the best p53 encoding pDNA nanosystems, the ability to promote protein expression was also evaluated. Overall, it was showed that CH polyplexes are more efficient on transfection when compared with the PEI polyplexes, resulting in higher P53 protein expression. Cells transfected with CH/p53-pDNA polyplexes presented an increase of around 54.2% on P53 expression, while the transfection with the PEI/p53-pDNA polyplexes resulted in a 32% increase.

## 1. Introduction

Gene delivery is a complex procedure where several obstacles must be overcome to successfully reach the target cell. Moreover, even after reaching the target cell, the biological effect is dependent on the effective delivery to the nucleus, for gene expression. Plasmid DNA (pDNA) has been considered a safer carrier for genes, but the pDNA itself must also be properly protected [1]. An ideal pDNA carrier should promote good cargo protection, excellent colloidal stability, a high cellular uptake, an efficient endosomal escape and finally, an effective nucleus import and DNA unpacking. Hence, it is extremely important to develop delivery systems that can reunite all these characteristics being also able to target the cell/tissue of interest, for instance by using a hydrophilic protective corona for minimizing the non-specific interactions [2]. 

Different approaches have been exploited for gene delivery. Naked DNA could be directly injected, but this approach would be limited to tissues reachable by direct injections (like skin or muscle) and also, it is unsuitable for systemic delivery due to the presence of serum nuclease [3], which would immediately degrade the therapeutic gene. On the other hand, viral vectors could be applied for transferring the genetic material into the host cells. These vectors are highly effective on both gene delivery and expression. However, the use of viral vectors has some drawbacks, as the possibility to provoke immune responses, the associated costs, the difficulty for the preparation, and the limit to just carry small amounts of genetic material [4]. 

Non-viral vectors have stood as safer alternatives to be applied for gene transfer. For the delivery of pDNA, systems are usually composed of cationic polymers or lipids with the ability to interact with the negatively charged DNA through electrostatic interactions leading to polyplexes and lipoplexes formation [5]. Non-viral vectors are usually safe (causing a low immune response), easily prepared, have a low production cost, and can be easily produced on large scale. Another important characteristic of these vectors is the ability to transfer different and large transgenes, being also able to be stored for long periods due to their stability [6]. 

Meanwhile, systemic delivery is a real challenge for these non-viral vectors since they need to survive in the bloodstream without being degraded or captured by cellular defence mechanisms. Also, when reaching the target tissue, the systems must go across the tissue and bind specifically to the target cells. After this internalization process, it is further required to surpass intracellular barriers such as the endosomal escape, the cytoplasm traffic, and finally, enter the nucleus [7]. So, the ability of non-viral vectors to overcome these barriers will dictate their efficiency. 

To develop pDNA-nanocarriers, different polymers have been applied. Linear or branched polyethyleneimine (PEI) has been extensively studied in a wide range of molecular weights, to promote the pDNA in vitro delivery [8,9]. However, some concerns regarding the high toxic behaviour of high molecular weight PEI (> 25 kDa) have limited its application. On the other hand, low molecular weight PEI demonstrated to be less toxic but showed poor transfection activity [10]. On the other hand, chitosan (CH), a natural cationic polymer that is a linear polysaccharide, has also been extensively used for several biomedical and pharmaceutical applications due to the advantageous properties, like biocompatibility and muco-adhesivity [11,12]. For instance, in 2011, Gaspar and co-workers developed a chitosan-based carrier to deliver the p53 encoding pDNA. With the use of this p53-CH delivery system, the authors were able to reinstate the levels of the P53 protein in cancer cell lines [12]. However, the CH polymer can also present some limitations regarding its application in gene delivery, due to the poor solubility in physiological conditions (pKa value around 6.3–6.4), which reinforces the need to proceed optimizing these systems.

Overall, in this research work, a comparative study was carried out to develop efficient pDNA nanocarriers using complexation between pDNA with different sizes and CH or PEI as cationic polymers. Therefore, the parameters for complexation between the selected polymers and pDNA were optimized, to find the most suitable conditions that led to improved systems, in terms of encapsulation efficiency, zeta potential, and size. Besides, this research work will enable the readers to access a full range of conditions that could be applied for both PEI and CH polyplexes formulation as well as will enable them to predict the kind of properties concerning the encapsulation efficiency, zeta potential, mean diameter, polydispersity, cytotoxicity, cellular uptake and protein expression that the systems can provide, considering the size of the target pDNA. Afterwards, the best polyplexes were characterized in a cellular environment, where a therapeutic nano-formulation based on a pDNA with a therapeutic target (in this case the p53 encoding pDNA) was used to investigate the ability to promote P53 protein expression.

## 2. Materials and Methods

### 2.1. Materials

The NZYtech Maxi Prep Kit was purchased from NZYTech (Lisbon, Portugal). The pcDNA3-FLAG-p53 Addgene plasmid 10838 (6.07 kbps) [13] and the p1321 HPV-16 E6/E7 Addgene plasmid 8641 (8.702 kbps) [14] were obtained from Addgene (Cambridge, MA, USA). The resazurin sodium salt, CH (Mw = 50–190 kDa) and all the reagents used in bacterial growth were obtained from Sigma-Aldrich (St. Louis, MO, USA). PEI (Mw = 10 kDa) was purchased from Polysciences. The DNA ladder was obtained from Bioline (London, UK). The DMEM-f12, EDTA, EGTA, Triton X-100, DMSO and the Fluorescein isothiocyanate (FITC) was purchased from Sigma-Aldrich, the Lipofectamine 2000 and the phenylmethylsulfonyl fluoride was purchased from Thermo Fisher Scientific (Lisbon, Portugal) and the Hoechst 33342^®^ was purchased from Invitrogen^TM^ Molecular ProbesTM (Carlsbad, CA, USA). P53 (human) ELISA kit was purchased from Enzo Life Sciences (Farmingdale, NY, USA). All reagents were of research-grade and used without further purification.

### 2.2. Methods

#### 2.2.1. Plasmids Production and Pre-Purification

The pcDNA3-FLAG-p53, the p1321 HPV-16 E6/E7 and the pVAX-GFP pDNA were amplified in *E. coli* DH5α. The growth was carried out at 37 °C, 250 rpm, in Erlenmeyer flasks with 250 mL of Terrific Broth medium (20 g/L of tryptone, 24 g/L of yeast extract, 4 mL/L of glycerol, 0.017 M KH_2_HPO_4_, 0.072 M K_2_HPO_4_) supplemented with 30 and 100 μg/mL of ampicillin for pcDNA3- FLAG-p53 and p1321 HPV-16 E6/E7, respectively. For pVAX-GFP, bacterial growth was performed by using 1 L shake flasks with 250 mL of Luria-Bertani medium (5 g/L yeast extract, 10 g/L tryptone and 10 g/L NaCl), supplemented with 30 μg/mL of kanamycin. The cells were grown until the log phase (OD600nm ± 9). Finally, the cells were collected by centrifugation and stored at −20 °C.

To obtain the pDNA, bacterial cells were disrupted by alkaline lysis and the different pDNA were pre-purified with the NZYTech kit, according to the supplier protocol to obtain the native pDNA (sc and oc isoforms). Briefly, after alkaline lysis, the pDNA was bound to the NZYTech anion exchange resin under appropriate low-salt and pH conditions. Then, the impurities were removed by a medium salt wash and, finally, the pDNA was eluted through the increase of ionic strength.

#### 2.2.2. Chitosan and PEI-Based Polyplexes Preparation

The preparation of pDNA-loaded polyplexes was performed using a complexation method through electrostatic interactions due to molar concentrations of positive charges present in the protonated amine groups of each polycation (N), and the negative charge of the phosphate groups of the DNA backbone (P). To determine the N/P ratio, the mass of one DNA phosphate group was used and, regarding the pH applied during the formulation of the polyplexes, it was also considered its anionic charge density near to 1.5, as reported by Mel’nikova and collaborators [14]. To the positive charges, the calculations performed used the pKa and the molecular weight of each polycation [15]. Then, 20 µg/mL of pDNA and 10 mg/mL of two different polycations (chitosan and PEI) were prepared in sodium acetate buffer (0.1 M sodium acetate/0.1 M acetic acid, pH 4.5). The desired polymer solution was individually prepared by the N/P ratio to be tested (the applied ratios varying between 0.1 and 50). After, 100 µL of the previously prepared ratios of the polymer solution was dropwise added, during 1 min, in 400 µL of the previously prepared pDNA solution, under stirring. The polyplexes were incubated at room temperature, for 15 min, and then recovered by centrifugation at 14,000 rpm, 10 min. The unbound pDNA was quantified using UV spectrometry and the encapsulation efficiency (EE) of at least three repetitions was determined through the following Equation (1):

EE% = (Total pDNA amount − pDNA supernatant amount)/(Total pDNA amount) × 100(1)

The different polyplexes were also characterized by zeta potential and hydrodynamic diameter. To perform this characterization, a Zetasizer Nano ZS particle analyser (Malvern Instruments, Worcestershire, UK), equipped with a He-Ne laser, at 25 °C was used, and the polyplexes were resuspended in ultrapure water. All the experiments were performed in triplicate and were analysed through Zetasizer software v 7.03 (Malvern Instruments, Worcestershire, UK). 

#### 2.2.3. Cell Culture and Transfection

Cell culture experiments were performed with the HeLa cervix cancer cell line and the non-malignant cell line, human dermal Fibroblasts (hFIB). To perform cell culture, DMEM-F12 medium supplemented with 10 % v/v heat-activated FBS and streptomycin (100 μg/mL) was used. Initially, cells were seeded in 25 cm^2^ T-flasks, at 37 °C, under a 5 % CO_2_ humidified atmosphere until confluence was reached. Afterwards, the cells were sub-cultivated by incubation on 0.18 % trypsin (1:250) with 5 mM EDTA. The in vitro transfection experiments were carried out by seeding 1 × 10^4^ cells/well in a 96-well plate with 200 µL of DMEM-F12 complete medium and incubated for 24 h. Then, a medium without FBS and antibiotic was used to facilitate transfection. The transfection of pDNA was performed with the previously prepared polyplexes. Briefly, the polyplexes were resuspended in 500 µL of Opti-MEM^®^ I medium, and a suitable volume containing 0.14 µg of DNA was applied in each well of the 96-well plates. The cells were incubated for a period of 6 h, and at this time point, the medium was exchanged to DMEM-F12 complete medium.

#### 2.2.4. Cytotoxicity

The cytotoxicity of different polyplexes was evaluated by using the resazurin assay. For this, HeLa and hFIB cells were seeded in 96-well plates, as described above. Resazurin (10 μL, 2.5 mM) was added to each well, two days after transfection. The plate was then incubated in the dark for 4 h, at 37 °C, in a humidified atmosphere of 5% CO_2_. After incubation, resofurin was measured using a plate reader spectrofluorometer (Spectramax Gemini XS, Molecular Devices, San Francisco, CA, USA), at an excitation/emission wavelength of λex = 560 nm and λem = 590 nm. Data represent the mean of three independent experiments.

#### 2.2.5. Cellular Uptake Analysis by Confocal Laser Scanning Microscopy (CLSM)

First, the pDNA biopharmaceuticals were labelled with FITC, allowing the follow-up of its cellular uptake and intracellular localization. Briefly, 5 µg of pDNA was added to 71 µL of labelling buffer (0.020 g of sodium (di)tetraborate in 1 mL of H_2_O) and 2 µL of FITC (100 mg of FITC in 200 µL of sterile DMSO). After this, the solution was stirred for 4 h at RT, protected from light. Finally, 85 µL of 3 M NaCl and 212.5 µL of absolute ethanol was added, to precipitate pDNA-labelled FITC by overnight incubation, at −20 °C. 

To evaluate pDNA-polyplexes cellular uptake kinetics, 1 × 10^3^ cells/well were seeded in complete DMEM-F12 in Ibidi µ-Slide 8-well cell culture treated chambers (Ibidi GmbH, Germany) and cultured overnight. Transfection was performed when 70 % of cellular confluence was achieved. Then, cells were incubated for 10 min with Hoechst 33342^®^ (1:1000) (Invitrogen^TM^ Molecular Probes^TM^) and, subsequently rinsed 3 times with PBS 1x (pH = 7.4). Transfection was performed for 1 h, with the different prepared pDNA-loaded polyplexes. Following the incubation period, the DMEM-F12 medium was exchanged and 4 % paraformaldehyde in PBS 1x was used for transfected cells fixation (for 20 min, at RT). To enable better visualization, transfected cells were washed three times with PBS 1x. Visualization was finally performed using a Zeiss LSM 710 laser scanning confocal microscope (Carl Zeiss SMT Inc., Jena, German) equipped with a plane-apochromatic 63×/DIC objective.

#### 2.2.6. P53 Expression by ELISA

The P53 ELISA kit (Enzo Life Sciences) was used to quantify the P53 protein after cells transfection with p53-pDNA vector. Briefly, following transfection with the CH and PEI polyplexes, HeLa cells were rinsed with ice-cold PBS 1x and homogenized in cell lysis buffer: 25 mM Tris-HCl buffer, pH 7.4; 2.5 mM EDTA; 1% Triton X-100; 2.5 mM EGTA; 25 mM phenylmethylsulfonyl fluoride and complete, EDTA-free protease inhibitor cocktail (Roche). Cell extracts were then centrifuged at 11,500 rpm for 7 min at 4 °C, and the supernatant was analysed using Bradford Protein Assay (BioRad) accordingly with the supplier’s instructions. Then, the protocol supplied by Enzo life Sciences for the ELISA was applied and the P53 protein expression was finally measured in a plate reader spectrofluorometer (Spectramax Gemini XS, Molecular Devices San Francisco, CA, USA) at 450 nm. Data represent the mean of two independent experiments.

#### 2.2.7. Statistical Analysis

Each experience was performed at least two or three times. Data are expressed as a mean ± standard error (S.D.). The statistical analysis performed was a one-way analysis of variance (ANOVA), followed by a multiple comparison test Tukey. A p-value below 0.05 was considered statistically significant. Additionally: * *p* < 0.05; ** *p* < 0.01; *** *p* < 0.001; **** *p* < 0.0001. Data analysis and statistical tests were performed in GraphPad Prism 6 software (San Diego, CA, USA).

## 3. Results and Discussion

### 3.1. Plasmid DNA Loading Efficiency in Polymer Nanoparticles

The encapsulation efficiency is one of the parameters that must be evaluated when it is intended for the selection and optimization of delivery systems designed to encapsulate, transport and release drugs or biomolecules. An ideal nanocarrier should be able to transport the highest amount of the target biomolecule possible, enabling its protection and delivery.

**Figure 1:**
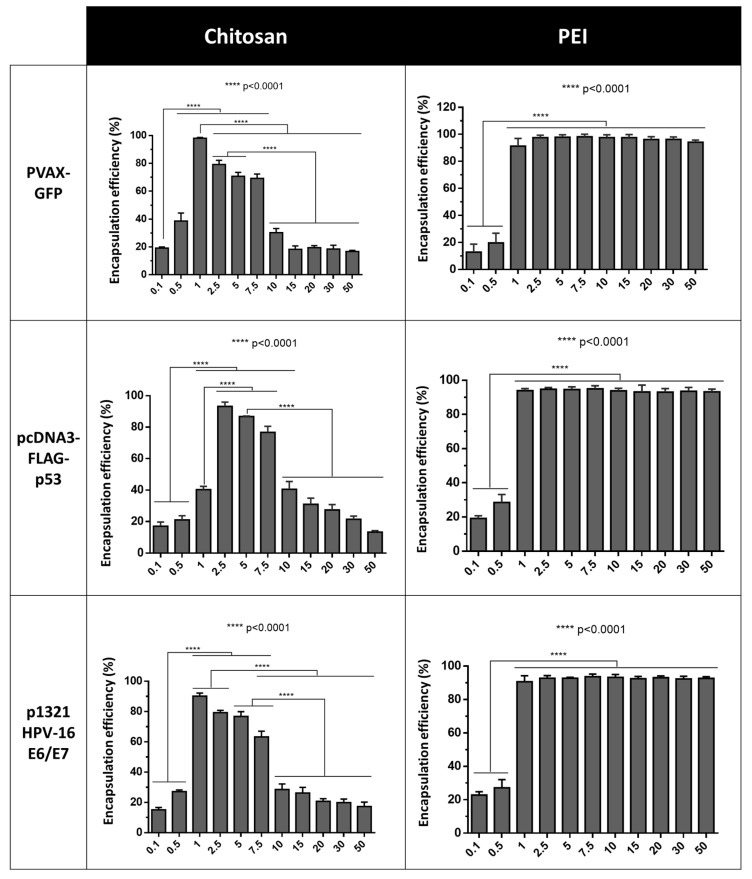
Encapsulation efficiency obtained for each formulation. A. CH-based nanocarriers; B. PEI-based nanocarriers. The asterisks indicate a statistically significant increase in the E.E. of the polyplexes produced with the different polymer ratio under study. Data are presented as mean ± S.D., *n* = 3.

In this study, to produce nanocarriers, the pDNA/polycation complexes were formed by electrostatic interactions between the negatively charged pDNA phosphate groups and protonated nitrogen atoms of the chosen polycations, as previously mentioned [16]. The work started with a screening of different pDNAs:CH/PEI ratios to evaluate which one could lead to higher pDNA encapsulation. The plasmids under study were pVAX-GFP, pcDNA3-FLAG-p53, and p1321 HPV-16 E6/E7 with the respective sizes of 3, 6, and 9 kbp. Through this screening, it was verified that the CH-based polyplexes presented higher encapsulation efficiencies for N/P ratios of 1 and 7.5 with the largest and smallest pDNA (Figure 1). It was also verified a tendency for an increased EE with the increase on N/P ratios until the highest EE value was reached (near 100% to pVAX-GFP and pcDNA3-FLAG-p53 and 90% to p1321 HPV-16 E6/E7), and then a sharp decrease of the EE was achieved for all the pDNAs. The lower EE were obtained for lower and higher polymer concentrations. In the case of higher ratios, the values obtained could be due to an excessive high positive charge causing a limitation on DNA access and interaction. Similarly, Gan and Wang (2007) have demonstrated that increasing the chitosan concentrations could lead to an increase in the solution viscosity which could be a major contributor to the decrease of the EE. Furthermore, the high viscosity associated with increased chitosan concentration hinders EE by deterring the biomolecules movement around chitosan molecular chain [17]. Concerning the low EE levels observed for the lower polymer ratios, it is suggested that the polymer was insufficient to induce the formation of polyplexes, due to the very low amount of available chitosan, limiting the interaction and encapsulation of pDNA.

Regarding the EE achieved for the PEI-based polyplexes, it was clear that for all the pDNA in the study, the best N/P ratios were above 1, and the maximum EE (very close to 100%) remained constant for all the higher N/P ratios (Figure 1). This result agrees with several studies that show the outstanding EE of PEI and its derivatives, regardless of the processing conditions. Despite their bio-applications, the high toxicity of PEI limits the efficiency of gene transfer in vitro and especially *in vivo* [15]. To fight this, several approaches have been reported to increase the gene transfection efficiency of PEI-based gene carriers while decreasing their intrinsic cytotoxicity [18].

### 3.2. Zeta Potential and Hydrodynamic Size Measurements

**Figure 2:**
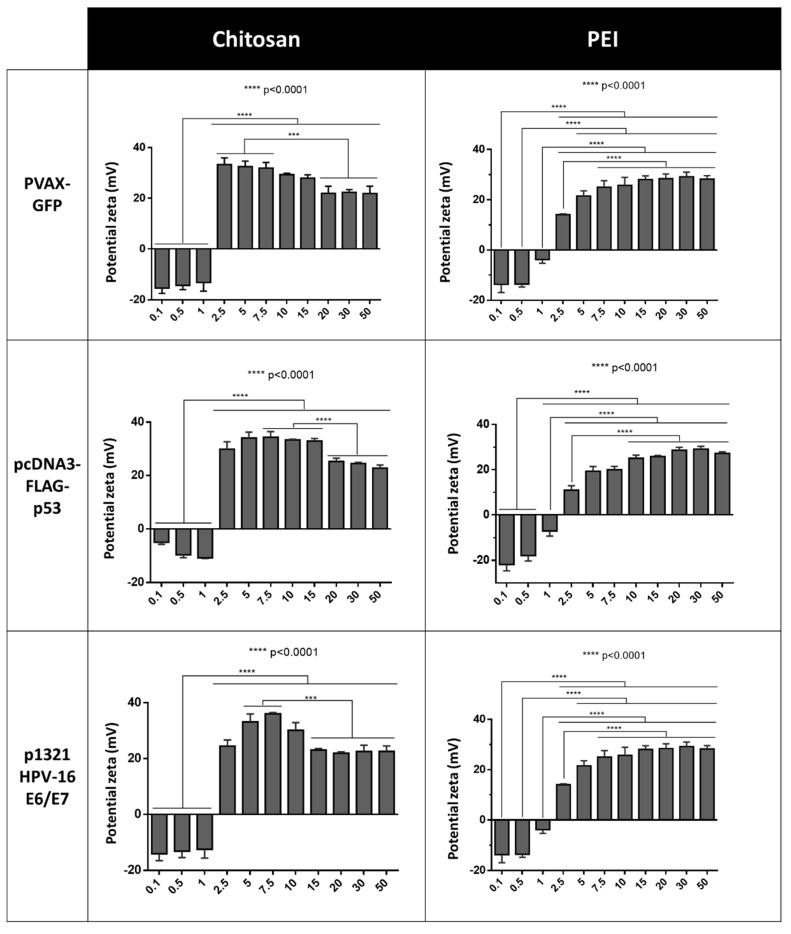
Zeta potential measurements for the different polyplexes. The asterisks indicate a statistically significant difference in the zeta potential between the polyplexes produced with the different polymer ratio studied. Data are presented as mean ± S.D., *n* = 3.

The positive surface charge is a very important requirement to be considered for any carrier to be used as an efficient gene delivery system. Moreover, the entry in the intracellular compartment will be simplified if the delivery system presents a small size (<200 nm) [16,17]. Thus, the zeta potential and size of the pDNA-loaded polyplexes were evaluated. The values obtained from the zeta potential measurements represent the value of the electrostatic potential at the plane of shear and, zeta potential values near ±30 mV are representative of stabilized particles [19]. Figure 2 shows the zeta potential values obtained for each pDNA conjugated with CH or PEI. Through the analysis of these results, it was possible to observe that for all the polyplexes, the zeta potential presented negative values up until the N/P ratio of 1. Meanwhile, the higher surface charge for CH-based polyplexes is comprised, in general, between N/P ratio 2.5 and 10 and, among these N/P ratios the desirable +30 mV was also accomplished. The highest value achieved was above 35 mV at N/P ratio 2.5 for the p53 encoding pDNA, and then the zeta potential decreased. It was also observed that for the pDNA with higher molecular weight (p1321 HPV-16 E6/E7), the decrease in the zeta potential was more evidenced for ratios above 7.5, while, for the smaller pDNA, a more pronounced difference was achieved only for N/P ratios higher than 15. For higher N/P ratios, the surface charge of the CH-based polyplexes suffered a slight decrease and then a stabilization. This decrease could hypothetically be related to a possible rearrangement in the polyplex structure, enabling a higher presence of the pDNA at the surface of the particles.

For PEI-based polyplexes, the highest zeta potential measurements were observed for higher N/P ratios when compared with CH-based polyplexes, being the values obtained very close to +30 mV. When the PEI values are closely analysed, it is observed a relationship between the ratios where it was found the stabilization of the EE and the maximum zeta potential. The zeta potential can also be used to determine whether a charged active material is encapsulated within the centre of the polyplex or on the surface. Thus, and considering the results obtained, it is suggested that the polyplexes produced present a “shell” made essentially of polymer being the genetic information fully protected inside the carriers. These polyplexes also presented a decrease in their zeta potential, but only for the highest N/P ratio used (N/P ratio of 50). This behaviour was previously described for aqueous solutions of two oppositely charged polyelectrolytes. In 2005, Zhang and Shklovskii showed that an increase in the polycation concentration ratio increases the size of the complexes reaching a maximum at the isoelectric point, and then decreases again accompanied by a “charge reversal” phenomenon [19,20]. To follow the characterization of the polyplexes, and considering the results achieved for the zeta potential and EE, the assessment of the hydrodynamic size was only performed for the best three ratios. Thus, for CH-based polyplexes, the N/P ratios of 5, 7.5, and 10 were selected, while the PEI-based polyplexes characterized were for the N/P ratios of 7.5, 10, and 15.

**Figure 3:**
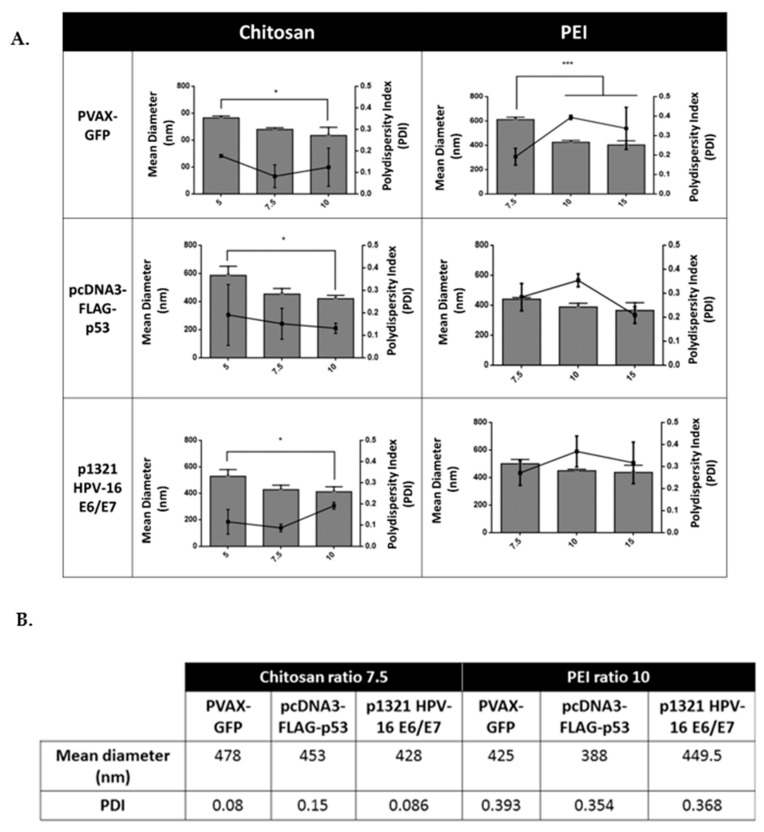
(**A**) Mean diameter (nm) and Polydispersity (PDI) values of the selected nanocomplexes; (**B**) Values obtained for the specific ratios chosen for the biological experiments. The asterisks indicate a statistically significant difference in the mean diameter between the polyplexes produced with the different polymer ratio studied. Data are presented as mean ± S.D., *n* = 3 (* *p* < 0.05 and *** *p* < 0.001).

As shown in Figure 3, the size of CH-based polyplexes decreased with the increase of the polymer ratio for pVAX-GFP and p53 encoding pDNA, while for p1321 HPV-16 E6/E7 the size decreased between N/P ratio 5 and 7.5, but then stabilized between N/P ratio 7.5 to 10. In the case of PEI-based polyplexes, de hydrodynamic size decreased between N/P ratio 7.5 to 10 and then stabilized between N/P ratio 10 to 15, for all the studied pDNA, but with a more pronounced effect for pVAX-GFP. Overall, it was found that the smaller nanocarriers were obtained at N/P ratio 15 and 10 for PEI and 7.5 and 10 for CH. Moreover, the increase of polydispersity, namely in the PEI-based polyplexes, could be induced by interactions through the accessible unbound DNA chains in one polyplex with the unbound PEI of another polyplex, resulting in some aggregation. On the other hand, polydispersity at relatively low ratios (as in the case of CH-based polyplexes) could be related to the initially formed polyplexes since they tend to agglomerate due to the nearly neutral surface charge of the polyplexes [21].

Overall, the PEI-based polyplexes showed to be smaller than the CH-based polyplexes, however, for the studied conditions, PEI polymer presented a very high PDI when compared with CH.

As mentioned above, the entry of large particles into the cell it is not easy, and their structure is not stable enough to resist the low pH environment and lysosomal enzymes [22]. Thus, the complete characterization and selection of suitable nanocarriers must include the evaluation of the biological response of the cells transfected with the polyplexes produced. In this case, the biological effect was assessed for the CH- and PEI-based polyplexes that presented the best values in terms of EE, Zeta potential, and hydrodynamic size. These conditions led to the selection of the N/P ratio of 7.5 for CH-based polyplexes and N/P ratio of 10 for PEI-based polyplexes.

### 3.3. Cytotoxicity Evaluation

**Figure 4:**
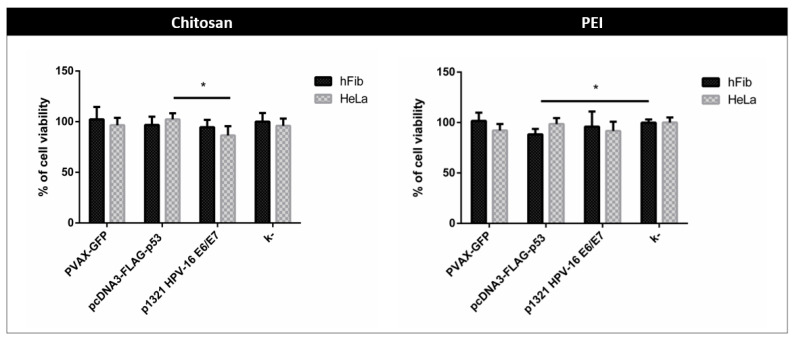
Evaluation of cell viability following transfection with CH and PEI polyplexes loaded with PVAX-GFP, pcDNA3-FLAG-p53 and the p1321 HPV-16 E6/E7 in different cell lines (human dermal fibroblasts (hFib) and HeLa cervix carcinoma), at 48 h after transfection. Data are represented as mean ± S.D., *n* = 3.

In vitro studies using cancer and non-cancer cell lines to predict human response, typically play a vital role in acquiring relevant information about the behaviour of these systems in a biological environment before further *in vivo* application of the formulations [23,24]. In this context, the toxicity levels of the formulations studied (PEI and CH nanoparticles) were accessed. The influence of the different polyplexes in HeLa cancer cells and human dermal fibroblasts (hFib) was studied and compared to search for the possible intracellular toxicity often associated with these cationic nanocarriers-mediated uptake. The results obtained (Figure 4) revealed that CH-based polyplexes loaded with different pDNA did not induce cytotoxicity, as the slight differences are not statistically significant in comparison to the negative controls. This was a predictable result since previous studies using pDNA-CH nanocomplexes have demonstrated low cytotoxicity for similar systems [12]. Therefore, this polymer has also been shown to destabilize the lipid bilayer, thus facilitating its cellular uptake which could be extremely advantageous for the efficient delivery of the desired genetic information [25].

Concerning the PEI-based polyplexes, statistical relevance was found for the cytotoxicity induced by p53-encoding pDNA-loaded polyplexes and the negative control but only for the hFib cells. This result could demonstrate potential *in vivo* toxicity of these carriers for non-tumour cells which could be a major concern in its future application [26]. This cytotoxicity presented by the PEI-based polyplexes could be explained by the higher polymer ratio applied. In fact, in literature, it was demonstrated that cationic polymers can induce cytotoxic effects at high concentrations because of their strong electrostatic interactions with the cell membrane, which can lead to destabilization and eventually rupture of the cell [27]. Also, some previous works made attempts to obtain information about the composition of the PEI-based polyplexes, demonstrating that large amounts of the PEI remain free and not involved in complex formation. These free PEI molecules presented higher toxicity than PEI bound as the polyplex [28]. Overall, these results seem to suggest that CH-based systems could be more promising for safer pDNA delivery than PEI-based formulations.

### 3.4. Evaluation of the Transfection Efficiency

Previous studies regarding the uptake mechanisms for CH or PEI nanoparticles indicated that CH-based polyplexes are mainly uptake through the clathrin-based endocytic mechanism, while PEI-based polyplexes have presented preference for the caveolar pathway in HeLa cells [29,30,31]. As referred, it is commonly accepted that complexes produced between DNA/cationic polymers are taken up by cells via endocytosis, however, further stages of their endosomal release, namely the transport along with the cytoplasm and also, the transfer to the nucleus and further DNA release, are less well understood. One of the mechanisms of the endosomal release of the complexes into the cytoplasm is based on the proton sponge hypothesis [32]. Research studies have demonstrated that several cationic polymers, such as PEI and CH, can buffer endosomal acidification causing an accumulation of protons which promotes an influx of chloride anions. This process increases osmotic pressure which promotes entry of water, and thereby the disruption of the endosome. It is worth mentioning that membrane destabilization by free polycation has been proposed as another mechanism that could also contribute to the endosomal release of the highly charged polymers [33].

**Figure 5:**
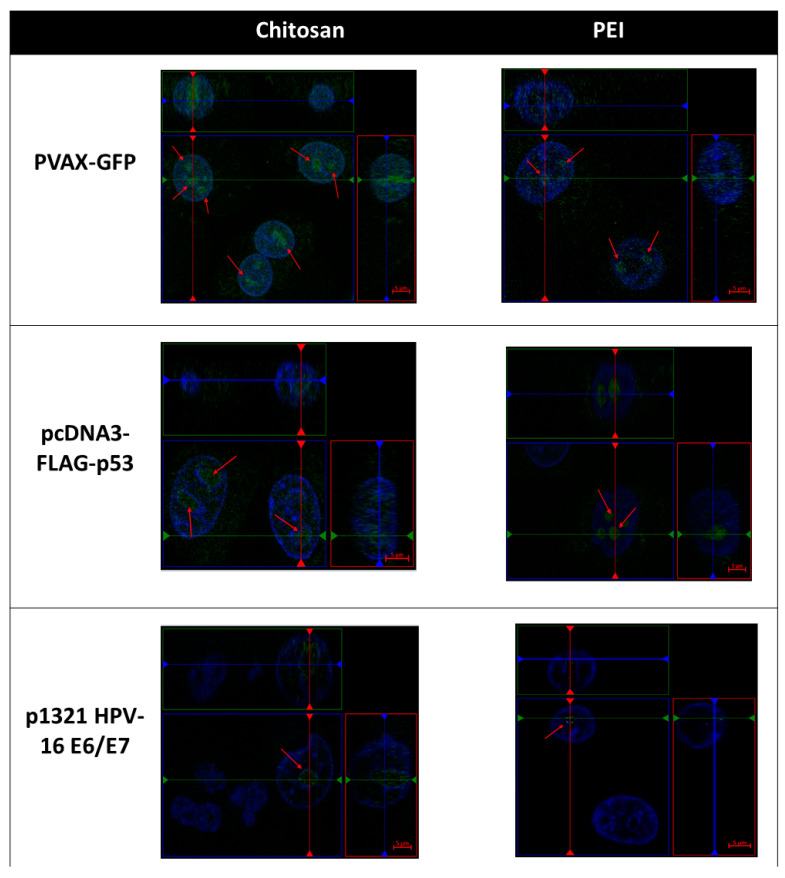
Orthogonal view of live cell imaging one hour after pDNA CH/PEI complexes addition to the HeLa cervix carcinoma cell line. The blue staining represents the nucleus of cells and the green staining represents FITC-labelled pDNA.

Concerning this, the DNA delivery in eukaryotic cells is a multistep process that begins with the condensation of DNA, the introduction of DNA into the systemic circulation, and targeted delivery to specific cells followed by cellular uptake, endosomal release, nuclear transport, and unpacking of the carrier/DNA polyplexes before the final step of translation in eukaryotic cells [3]. In the present work, to explore the DNA delivery process, the behaviour of these molecules was monitored by confocal laser scanning microscopy. The cell live imaging after 1 h of transfection (Figure 5) showed the entry into the cell for all the pDNA/cationic polymers studied. The carriers of smaller pDNA promoted faster cell transfection than the ones delivering the largest pDNA, for both CH- and PEI-based polyplexes. The system that seems to be faster for transfection was the CH with pVAX-GFP. In this case, cells presented a large number of labelled-particles inside the nucleus, which can be indicative of the efficiency of these systems entering the nucleus. Through these results, it is predictable that CH-based polyplexes could be more efficient on transfection when compared with the PEI-based polyplexes, which is also following the previous cytotoxicity results.

Literature mentioned that lower transfection levels at higher polymer ratios may be induced by competition between the excess of cationic polymer present in the formulation. This extra polymer can bind to the cell surface, preventing complexes from being efficiently internalized [27]. This explanation about the excess of the polymer could be the reason for PEI nanoparticles present a slightly higher cytotoxic behaviour as well as a lower transfection rate since for this case a higher ratio is applied when compared with the CH ratio chosen. Another reasonable explanation could be a slight difference in the zeta potential presented for both carriers. The CH-based polyplexes presented the highest mV value and this could also favour the interaction with the negative cell membrane. 

Concerning the largest pDNA studied, the behaviour for CH- and PEI-based polyplexes was very similar, since in both cases cells presented a low transfection rate and when transfected, few labelled-particles were found in the nucleus, which can suggest that low protein expression could be achieved. Different studies have already been performed to compare the influence of the pDNA size on the transfection effectiveness, being verified that actually, as short as the pDNA is, as higher is the transfection rate. Kreiss and collaborators (1999) hypothesized, based on the scientific evidence from their research work, that the nuclear entry via the nuclear pores might be dependent on plasmid size (as shorten was the pDNA as effective was the gene transfer process) [34].

### 3.5. P53 Protein Expression

The formation of these polymeric nanoparticles occurs by the entrapment of the genetic material into the polymer matrix and thus, the release rate of pDNA is dependent on cationic polymer biodegradation. Several studies have suggested that DNA unpacking is one of the major intracellular barriers to effective expression [35]. It has been recognized that a balance between DNA protection and its ability to dissociate from the nanoparticles must be achieved to obtain efficient protein expression [27].

**Figure 6:**
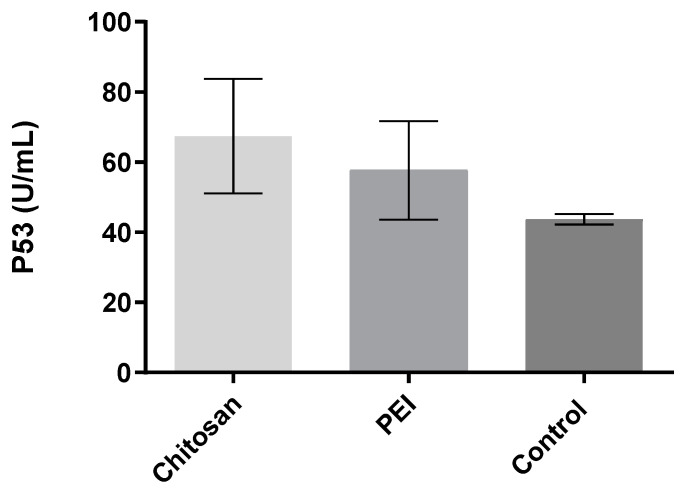
Evaluation of p53 protein expression following administration of p53-pDNA polyplexes of CH and PEI in HeLa cervix carcinoma. Data are represented as mean ± S.D., *n* = 2.

To evaluate the real ability of these polyplexes to transfer genetic information across the cell to reach the nucleus, HeLa cells were transfected with both polyplexes conjugated with the p53 encoding pDNA, and then P53 protein levels were measured through an ELISA analysis. The cell line was previously used to assess the expression of P53 after a transfection experiment and showed to be very sensitive to the p53 encoding gene treatment [33,36]. From the analysis of Figure 6, it is possible to observe that, although the chosen cells already present a basal expression of the P53 protein, when transfected with CH-p53 encoding pDNA polyplexes, this level increases around 54.2%, and when transfected with PEI-loaded polyplexes with the same pDNA, the protein level increases around 31.96%, in comparison with the P53 basal levels. 

As mentioned, the free polycation in the DNA/polymer nanoparticles dispersion has shown to be mandatory to an efficient transfection, since it helps on the endosomal scape. Some studies have shown that DNA/polycation complexes alone cannot trigger their endosomal escape through the proton sponge mechanism without a sufficiently high content of free polycation inside the endosome. When nanoparticles find the mechanism to escape from the endosome, the DNA released from the polycation migrates into the nucleus for further transcription. However, it was also described that gene expression decreases when DNA was either tightly or loosely bound to the polycations, such as PEI or CH [35,37]. The tight bound and highly stable polyplexes will be readily endocytosed but possibly not disassembled to access the transcription machinery. On the other hand, DNA weakly bound to the polycation will produce complexes that will dissociate prematurely in the medium and not even be endocytosed by cells [38].

Regarding our results from confocal microscopy analysis, it was possible to observe a slight increase of the p53 nanoparticles entrapment for CH-based polyplexes, a behaviour that could suggest a lower P53 protein expression for the PEI-based polyplexes. Besides this, we can also suggest that the decreased protein expression could be related to a weak linkage of DNA with PEI, when compared with the CH/DNA polyplexes, which could lead to rapid degradation of the delivered gene. This degradation process decreases the amount of viable genes to be used for the transcription machinery, which will also decrease the amount of the final P53 protein produced. 

It is also important to refer that using the DNA polymer complexation method, we were able to accomplish better P53 protein expression using the CH-based polyplexes than Gaspar and co-workers (2011). In that previous study, an ionotropic gelation technique was performed between CH and TPP (used as polyanion) and where the p53 encoding pDNA vector was also added. To accomplish the production of the nanocapsules, TPP+pDNA was dropwise added to the chitosan solution. However, using this method the authors only accomplish 40% of P53 expression [12]. 

Concerning all the above mentioned, an effective gene delivery nano complex should provide an appropriate balance between the DNA and polycation, to promote not only binding strength and stability but also, guarantee that DNA can dissociate intracellularly for gene expression.

## 4. Conclusions

To promote gene transfer, non-viral systems such as nanoparticles have arisen as efficient and safe delivery tools. Several cationic polymers have been used in the nanocarriers development, and in this work, different pDNA were conjugated with CH and PEI to search for the suitable nano complex combination. Regarding the surface charge and encapsulation efficiency characterization, it was verified that, depending on the ratios used, it could be possible to define suitable conditions to prepare positively charged nanocarriers highly efficient on DNA loading (around 80 to 100% for the best conditions), which is crucial for the application. Additionally, the biological effect was assessed for the CH- and PEI-based polyplexes that presented the best properties in terms of EE, Zeta potential and hydrodynamic size (N/P ratio 7.5 for CH and N/P ratio 10 for PEI). Through the cytotoxic profile, it was observed biocompatibility for CH-based polyplexes, however, for p53-encoding pDNA/PEI polyplexes it was verified slight toxicity in normal cells which could be a handicap for future therapeutic application of these polyplexes. Regarding the transfection efficiency, it was verified that all the pDNA /cationic polymers systems studied were able to transfect the cells, but a higher efficiency was reached for the smaller pDNA. Finally, the ability of the polyplexes to promote P53 protein expression was also addressed using HeLa cancer cells. From the results obtained, the P53 levels increased around 54.2% and 32% when CH- and PEI-based polyplexes were respectively applied.

Overall, it is possible to refer that an effective gene delivery nano complex should provide an appropriate balance between the DNA and the polycation. The chosen conditions for the polyplexes formulation should consider parameters such as EE, size, zeta potential and polydispersity being also needed to perform a careful polymer selection. Besides, given the presented results and the used complexation conditions it was observed that the polyplexes using an N/P ratio 7.5 of CH presented less cytotoxicity, were more efficient regarding cell uptake and finally led to an increased protein expression when compared with the PEI polyplexes, which suggests that CH polyplexes can be more suitable for pDNA delivery.

## Data Availability

The data presented in this study are available on request from the corresponding author.
